# Multi-Omics Tumor Immunogenicity Score Predicts Immunotherapy Outcome and Survival

**DOI:** 10.3390/biology14121698

**Published:** 2025-11-28

**Authors:** Axel Gschwind, Nadja Ballin, Alexander Ott, Andrea Forschner, Amelie Knapp, Öznur Öner, Michael Bitzer, Ghazaleh Tabatabai, Andreas Hartkopf, Thorben Groß, Markus Reitmajer, Christopher Schroeder, Stephan Ossowski, Sorin Armeanu-Ebinger

**Affiliations:** 1 Institute of Medical Genetics and Applied Genomics, University of Tübingen, 72076 Tübingen, Germany; 2 Institute for Bioinformatics and Medical Informatics, University of Tübingen, 72076 Tübingen, Germany; 3 Center for Dermatooncology, Department of Dermatology, University of Tübingen, 72076 Tübingen, Germany; 4 Center for Personalized Medicine Tübingen, University of Tübingen, 72076 Tübingen, Germany; 5 Center for Neurooncology, University of Tübingen, 72076 Tübingen, Germany; 6 Department of Women’s Health, University of Tübingen, 72076 Tübingen, Germany; 7 Department of Medical Oncology and Pneumology, University of Tübingen, 72076 Tübingen, Germany

**Keywords:** tumor immunogenicity, immune checkpoint inhibitors, immunotherapy, anti-PD1, multi-omics, next-generation sequencing, whole exome sequencing, bulk RNA sequencing

## Abstract

Background: Tumor immunogenicity is a concept for modeling the susceptibility of tumors to immune checkpoint inhibitors (ICIs) and other immunotherapies. Single biomarkers, such as tumor mutation burden (TMB) or *PDL1* expression, have been shown to correlate with ICI outcomes but are poor predictors of overall and progression-free survival (OS, PFS). Complex machine learning models that integrate multiple biomarkers have shown improved predictions but often lack clear a priori interpretability. In this study, we developed a coherent Multi-Omics Tumor Immunogenicity score (MOTIscore) that combines immunogenicity biomarkers derived from genomic and transcriptomic data and demonstrated its generalizability across multiple cancer types. Methods: Several immunogenicity biomarkers, including TMB, neoantigen burden, T-cell receptor repertoire, *PDL1* expression, *B2M* expression, and variants in pathways of ICI response and resistance, were integrated using a weighted sum scoring scheme. The weights were determined using statistical tests in a large melanoma ICI cohort. We compared the MOTIscore with a machine learning (ML) model trained using the same biomarkers and evaluated the model using melanoma, gastric cancer, and pan-cancer datasets. Results: MOTIscore achieved results similar to those of the ML model in predicting ICI in melanoma and gastric cancer, with both outperforming TMB. Gastric cancer and melanoma patients with high MOTIscores had a significantly extended overall and progression-free survival. Gene set enrichment analysis revealed the enrichment of immune-related pathways in patients with high MOTIscores. Differential expression analysis between patients with high and low immunogenicity identified highly expressed C-X-C motif chemokine ligands as important characteristics associated with successful ICI therapy and significantly improved PFS. MOTIscores varied widely across cancers treated in the molecular tumor board at our hospital and showed distinct distributions between non-immunogenic and immunogenic cancer types. Conclusions: MOTIscore demonstrated improved ICI outcome predictions compared to single-omics biomarkers. Patients with higher tumor immunogenicity also show significantly improved OS and PFS in melanoma and gastric cancer. The results demonstrate the potential use of the MOTIscore to prioritize ICI in personalized cancer treatment. However, ICI outcomes and survival should be investigated in prospective studies, and additional cancer types and larger patient cohorts are needed.

## Simple Summary

Tumor immunogenicity is the ability of cancer cells to evoke an immune response. Several tumor properties have been associated with immunogenicity. However, only a few single biomarkers are used for clinical decision making, providing a fragmented view of the complex tumor-immune interactions. In this study, we propose a multi-omics tumor immunogenicity score (MOTIscore) that combines multiple biomarkers to improve the characterization of tumor immunogenicity. It integrates several biomarkers extracted from DNA and RNA sequencing data and applies weighted sum scoring. The MOTIscore integrates various biomarkers, including tumor mutation burden and immune cell infiltration. We evaluated MOTIscore as a predictor of the outcomes of state-of-the-art cancer immunotherapy. It was predictive of therapeutic success in both skin and gastric cancers, thereby outperforming the well-established tumor mutation burden and a machine learning model. The MOTIscore may help consider immunotherapy approaches for patients discussed in molecular tumor boards.

## 1. Introduction

Tumor immunogenicity plays an important role in state-of-the-art cancer treatment using immune checkpoint inhibitors (ICIs). Immunogenicity reflects the ability of cancer cells to be recognized and attacked by the immune system [1]. Various biomarkers, such as tumor mutation burden (TMB) and immune cell infiltration, are associated with tumor immunogenicity but provide only a fragmented view of the tumor immune microenvironment. The complex nature of the immune-tumor interface cannot be fully captured by a single biomarker and requires a holistic approach that takes biomarkers from multiple sources into consideration [2]. In line with this, most single-omics biomarkers have shown poor efficacy in predicting ICI treatment outcomes in several cancer types. For example, *PDL1* expression, quantified as Combined Positive Score (CPS) or Tumor Proportion Score (TPS), is an approved and commonly used immunohistochemical biomarker for the selection of patients for ICI. However, neither score is sufficiently selective for ICI [3,4]. Thus, many patients with low CPS or TPS respond well to ICI, whereas many patients with high CPS or TPS do not respond. This underscores the urgent need for the identification of more reliable ICI biomarkers and composite immunogenicity models to predict therapy response with higher accuracy [5].

Next-generation sequencing (NGS) of both tumor DNA and RNA can be employed to extract a diverse set of genomic and transcriptomic biomarkers that characterize immunogenicity [6]. Such biomarkers include, in addition to TMB and immune cell infiltration, several tumor genome-intrinsic factors, such as HLA genotypes or mutations in genes associated with ICI resistance. Biomarkers derived from whole-exome sequencing (WES) and bulk RNA sequencing (RNA-Seq) are thought to provide orthogonal and complementary information [7]. For example, information about mutational processes generating neoantigens can be retrieved from WES data, whereas RNA-Seq captures orthogonal transcriptional data, such as B- and T-cell repertoire and immune cell infiltration. Integrating several such biomarkers into a multi-omics immunogenicity model has the potential to improve our understanding of the complex tumor-immune interface, thereby improving ICI outcome prediction [8].

Several methods have been proposed to combine multi-omics biomarkers for a comprehensive description of tumor immunogenicity, including statistical models and machine learning algorithms [9,10]. However, machine learning models often lack explainability and interpretability, which are important criteria for clinical usability [11]. The utilization of advanced algorithms as black boxes makes it difficult for clinicians to trust and understand how these algorithms reach their conclusions. Popular explainability techniques, such as Shapley Additive exPlanations (SHAP) or Local Interpretable Model-Agnostic Explanations (LIME), provide post hoc interpretations of the models [12]. However, these explanations can vary widely among patient cohorts, limiting their reproducibility and potential use in clinical settings.

Here, we propose a straightforward Multi-Omics Tumor Immunogenicity score (MOTIscore) that integrates several biomarkers derived from WES and RNA-Seq data. Both types of sequencing data are becoming increasingly available as part of routine personalized cancer treatment strategies. The MOTIscore model has a transparent design and can be interpreted a priori by providing the exact impact of each feature on the immunogenicity estimate. These are crucial prerequisites for potential clinical applications [13]. The MOTIscore leverages several biologically meaningful immunogenicity biomarkers, the selection of which was manually curated. It includes a comprehensive list of eight initial biomarkers: TMB [14], neoantigen burden [15], T-cell receptor (TCR) repertoire [16], ICI-resistance mechanisms [17,18], ICI-response mechanisms [19], HLA evolutionary divergence (HED) [20], *PDL1* expression [21], and *B2M* expression [18]. The model applies a weighted sum scoring scheme and determines the weights assigned to each biomarker using basic statistical techniques, such as Fisher’s exact test or the Mann–Whitney U test [22].

While MOTIscore was designed as an interpretable methodology for integrating multi-omics biomarkers, this work also investigates its practical oncological relevance. We developed and validated the MOTIscore using publicly available cohorts of cancer patients who received ICI treatment, including those with melanoma and gastric cancer. The sequencing and clinical data provided by these studies were well-documented and all cases were annotated with ICI outcomes. An additional validation cohort comprised real-world patients with various cancers from the local molecular tumor board (MTB) of the University Hospital Tübingen in Germany [23]. The MTB patients were treated with various anti-cancer strategies, of which immunotherapy was rare due to a lack of robust biomarkers for the selection of patients for ICI. Hence, by introducing MOTIscore into MTB diagnostics, we aim to identify immunogenic tumors that are likely to benefit from ICI therapy. The use of multiple cohorts for evaluation allowed us to explore the generalizability of the MOTIscore model across various cancer types.

The evaluation of the MOTIscore proved its general ability to discriminate ICI responders from non-responders, thereby outperforming the traditional biomarker TMB. ICI-treated patients with high MOTIscores showed improved survival both in terms of progression-free survival (PFS) and overall survival (OS) in gastric cancer and melanoma. This suggests that the MOTIscore could facilitate improved selection of patients for ICI treatment. To compare the performance of the MOTIscore with a machine learning (ML) approach, we trained a multivariable least absolute shrinkage and selection operator (LASSO) regression model using the same set of input biomarkers. Both models achieved similar predictive performances. Furthermore, a gene set enrichment analysis (GSEA) revealed the role of highly expressed C-X-C motif chemokine ligands as predictive biomarkers for ICI outcomes and survival, thereby increasing the number of impactful features for immunogenicity models.

## 2. Materials and Methods

Our multi-omics model integrates multiple immunogenicity biomarkers derived from WES and RNA-Seq data into a weighted sum score. A flowchart of the model is provided in Supplementary Figure S1.

### 2.1. Initial Biomarkers

We derived genomic and transcriptomic biomarkers from paired tumor-normal WES and corresponding RNA-Seq tumor samples. All biomarkers have been associated with tumor immunogenicity and ICI response in previous studies. In detail, we curated the following initial list of immunogenicity biomarkers:

- Tumor Mutation Burden (TMB) [14]
- Neoantigen burden [15]
- T-cell receptor (TCR) repertoire [16]
- ICI-resistance mechanisms: *B2M* mutations and defects of the antigen processing machinery (APM), which includes the genes *TAP1*, *TAP2*, *TAPBP*, *CIITA*, *CALR*, and *CALX* [17,18]
- ICI-response mechanisms: mutations in *LRP1B* [19]
- HLA evolutionary divergence (HED) [20]
- *PDL1* expression (in counts per million (CPM)) [21]
- *B2M* expression (in CPM) [18]

We provide bioinformatic implementation details for each biomarker in Section 2.5.

### 2.2. Biomarker Selection and Weight Determination

We selected biomarkers and determined the weights for the MOTIscore using a cohort of melanoma samples for which both ICI treatment outcomes and sequencing data were available. The melanoma discovery cohort was assembled from six previously published melanoma studies. We defined binary outcomes as responders and non-responders according to the Response Evaluation Criteria in Solid Tumors (RECIST) [24]. Patients with complete and partial responses were classified as responders, whereas those with stable and progressive disease were considered non-responders.

To test whether a biomarker was significantly enriched in one of the outcome groups, we used Fisher’s exact test for categorical biomarkers, and Mann–Whitney U test for continuous biomarkers. Biomarkers that did not show a significance level of at least *p* < 0.05 were excluded from MOTIscore. We then checked all biomarkers for mutual correlations using Spearman’s correlation coefficient ρ. If two biomarkers showed a correlation coefficient of ρ > 0.5, the biomarker with the smallest effect size was excluded.

The weights assigned to each biomarker were calculated as log_10_ *p*-values, which were normalized by the sum of all weights of the biomarkers that were selected for the MOTIscore. Finally, we calculated the score as the weighted sum of the z-scores of the selected biomarkers.

### 2.3. Bioinformatics Pipeline

All tumor-normal WES and RNA-Seq samples were analyzed using the megSAP pipeline, available at GitHub https://github.com/imgag/megSAP (commit b148ff9, accessed on 12 September 2024). For the WES samples, the reads were aligned with Illumina DRAGEN 4.2.4 against GRCh38 [25]. Subsequently, DRAGEN was used as a somatic variant caller. All detected somatic variants were annotated using various tools and databases, including ensemble variant effect predictor (VEP) v112 and the Catalogue of Somatic Mutations in Cancer (COSMIC) v99 [26,27]. ClinCNV was used to identify somatic CNVs [28]. For the RNA-Seq samples, megSAP utilized STAR 2.7.11b for the alignment of the reads [29]. Read counts were estimated using subread featureCounts 2.0.6 and normalized to CPM [30]. After executing the basic analysis steps of megSAP, we derived additional biomarkers. For Implementation details, see that section.

For subsequent analyses of gene expression, we used the R package DESeq2 1.46.0 to detect differentially expressed genes (DEGs) and clusterProfiler 4.14.6 for gene set enrichment analysis (GSEA) [31,32].

### 2.4. Quality Control

We excluded every sample in this study that did not meet a minimum coverage of 60× for the tumor and 20× for the normal sample. In addition, we discarded all samples that showed an insufficient correlation of <80% between the SNPs of the tumor and normal samples, indicating potential sample confusion. If more than 70% of a sample’s somatic variants appeared in gnomAD, we assumed sample contamination and excluded these samples from the cohort. As an additional quality filter, all samples with a tumor content of <30% were excluded from the study. The tumor content was determined using ClinCNV, and if no CNVs were detected using the median of the 15 highest variant allele frequencies (VAFs). Samples with zero detected somatic variants were visually inspected in IGV and discarded if the tumor content was below 30%.

At the level of SNVs and small indels, we required a minimum read depth of 20× for both the tumor- and normal samples. Only somatic variants with a VAF > 5%, which were present in at least three distinct sequencing reads, were considered. For somatic CNVs, we specified a minimum loglikelihood of >100. All CNV calls were inspected manually and the ClinCNV baselines were corrected if necessary.

### 2.5. Implementation Details

#### 2.5.1. Target Region

The results presented in this study originated from different sequencing centers and enrichment kits. A common intersecting target region was constructed to avoid batch effects. In short, we determined the region that was covered at least 20× for each normal sample and cohort. We included every base with a coverage of 20× in at least half of the samples in the target region of a specific cohort. This study-specific region was then intersected with the coordinates of all exons (±2 bases). Subsequently, the common target region was obtained by intersecting all study-specific target regions. This intersection was used for the analysis of all samples and subsequent biomarker extraction, with the exception of CNV calling, which required cohort-specific target regions.

#### 2.5.2. Tumor Mutation Burden

We calculated TMB as the total number of nonsynonymous somatic variants, normalized by the size of the target region in megabases. To avoid batch effects, we only considered somatic variants that lied in the intersecting exome target region. All variants with a predicted VEP impact higher than “protein altering variant” were considered as nonsynonymous.

#### 2.5.3. Neoantigen Burden

We used pVACseq 4.4.1, a wrapper pipeline integrating several neoantigen prediction algorithms, for the prediction of neoantigens [33]. The pipeline was executed using three algorithms: NetMHCpan, NetMHC, and MHCflurry. Every predicted neoantigen candidate with a predicted median binding affinity of <500 nM was subsequently considered for the calculation of the neoantigen burden. As an additional filtering step, we excluded all neoantigen candidates that were not expressed in at least one sequencing read of the RNA-Seq data. If deletions of HLA alleles were detected in a sample (see Section 2.5.7, we considered only those neoantigen candidates that bound to the remaining allele.

#### 2.5.4. TCR Repertoire

TCR clones were extracted from the RNA-Seq data using TRUST4 v1.1.4 [34]. For further analysis, we included only clones that were detected at least twice in a sample. Subsequently, we used the number of distinct TCR α chain clones to calculate the Shannon entropy *H*:

$$H=\;-\sum_{i=1}^{N}p_{i}\log p_{i}$$

where *p_i_* represents the relative frequency of the TCR clone *i*. *N* is the total count of the detected TCR clones. The TCR Shannon entropy serves as an indicator of TCR diversity in the sample. Therefore, it can be used as a proxy for the TCR repertoire and tumor infiltrating lymphocytes.

#### 2.5.5. ICI-Resistance Mechanisms

We defined a binary biomarker that describes alterations in known ICI resistance genes. This biomarker was set to −1 if any deleterious variant occurred in the resistance mechanisms and 0 otherwise. The negative sign reflects the direction of the mutated resistance mechanism, as it leads to lower immunogenicity. We included any deleterious alterations that affected the antigen processing machinery genes, *TAP1*, *TAP2*, *TAPBP*, *CIITA*, *CALR*, *CALX*. In addition, we considered deleterious alterations of *B2M* [35].

Only homozygous deletions and variants with a high VEP impact or annotated COSMIC Cancer Mutation Census (CMC) levels in any of these seven genes were considered deleterious. For any alterations, we also required that they affected the main clone. For CNVs, we determined this directly from the ClinCNV calls. For the SNVs and indels, this was achieved by filtering out any variant with a variant allele frequency of less than 80% of the tumor clonality.

#### 2.5.6. ICI-Response Mechanisms

*LRP1B* mutations are associated with higher immune cell infiltration and have recently been shown to improve ICI outcome [19]. We considered any somatic alteration in *LRP1B* with high VEP impact and CMC annotation as deleterious, as well as any homozygous deletion. That is, we excluded variants of unclear significance. We considered all mutations regardless of their clonality. If a mutation was present, this biomarker was set to 1, and 0 otherwise.

#### 2.5.7. HLA Evolutionary Divergence

HLA evolutionary divergence (HED) measures the evolutionary protein distance between two HLA alleles [36]. In this study, we first determined HLA genotypes using our in-house implementation of hla-genotyper, available at GitHub https://github.com/axelgschwind/hla-genotyper (release tag 2022_05, accessed on 24 April 2025) [37]. We then examined whether there was an overlapping CNV that led to the loss of one HLA allele with a CNV clonality of at least 25%. As an additional check, we examined the ratio of assigned hla-genotyper read counts between the tumor and normal samples. We also assumed that an HLA allele was lost if there was a deviation of >40% in the read count ratios of the tumor and normal samples. If one HLA allele was lost, similar to homozygosity, HED was set to 0. HED between the two detected alleles was then measured using Grantham distance, a protein distance measure that integrates several physicochemical properties [38]. We determined HED only for the HLA binding grooves and not for the whole amino acid chain.

#### 2.5.8. Gene Expression

Read counts were quantified using the RNA-Seq megSAP pipeline. Subsequently, the expression of the immune-related genes *PDL1* and *B2M* was quantified as counts per million (CPM) [18,21]. For the assembled ICI melanoma cohort, we corrected gene expression for batch effects using pyCOMBAT 0.3.3 [39].

## 3. Results

In this study, we integrated several immunogenicity biomarkers derived from WES and RNA-Seq data into a weighted sum score model. The weights assigned to each biomarker were determined using statistical tests (Fisher’s exact test and Mann–Whitney U test) comparing two groups (RECIST responders vs. non-responders) of the melanoma meta-cohort with available ICI outcome data. Biomarkers that did not meet the minimum significance threshold of *p* < 0.05 were excluded. Features with high mutual correlation coefficients were filtered to avoid bias in the score weights. Subsequently, we calculated the MOTIscore using z-standardized biomarkers. The score was then evaluated using three distinct cohorts, two of which had annotated ICI response data. Supplementary Figure S1 shows an outline of the proposed approach. MOTIscore was benchmarked against a LASSO model using the same set of biomarkers. The methodology of the LASSO model has been described elsewhere [9].

### 3.1. Cohorts

We developed and evaluated MOTIscore using three distinct datasets. Two of these cohorts, melanoma (*n* = 225) and gastric cancer (*n* = 33), were assembled from public studies and evaluated using ICI outcome data. The third, pan-cancer MTB cohort (*n* = 559), had only partially annotated ICI outcome data and represents heterogeneous cancer patients from the local molecular tumor board of the University Hospital Tübingen, Germany. In the MTB cohort, we identified samples with very high MOTIscore to consider patients with highly immunogenic tumors who could potentially benefit from ICI therapy.

In detail, the melanoma cohort (*n* = 225) was assembled from six studies for which pre-ICI-treatment sequencing data (WES and RNA-Seq) were publicly available: Amato et al., Hugo et al., Liu et al., Pyke et al., Riaz et al., and Wolchok et al. [40,41,42,43,44,45]. A total of 27 samples were excluded based on our quality control criteria. This resulted in a discovery cohort of 225 ICI-treated melanoma patients for whom paired tumor-normal WES and bulk RNA-Seq data were available. According to the RECIST classification, 98 patients were classified as responders and 127 as non-responders, respectively.

The ICI gastric cancer dataset (*n* = 45) was published by Kim et al. with publicly available pre-ICI-treatment sequencing data [46]. After quality control with 12 removed samples, this dataset resulted in 33 ICI-treated patients with gastric cancer, of whom seven were responders and 26 were non-responders.

The MTB cohort consisted of patients who sought medical advice at the MTB in Tübingen between 2020 and 2024 and who provided informed consent to use their sequencing data for clinical research. After quality control, which removed 91 samples, the MTB cohort comprised 559 patients. It contains several cancer types, with brain tumors and melanomas being the most frequent. It comprises sequencing data at various time points, that is, pre- and post-treatment samples. The patients included in the MTB cohort received various anti-cancer drugs, including ICIs. An overview of the MTB cohort and cancer types is provided in Supplementary Table S1.

### 3.2. Selection of Biomarkers and Weight Determination

We determined the weights for the MOTIscore weighted sum scoring model using the ICI melanoma cohort of 225 patients, of whom 98 were responders and 127 were non-responders. Five biomarkers remained after filtering out features that did not meet the minimum significance level of *p* < 0.05 for discriminating responders from non-responders. TMB, neoantigen burden, mutations in response pathways, TCR repertoire, and *PDL1* expression were retained as biomarkers for the MOTIscore. A subsequent correlation analysis revealed that TMB and neoantigen burden were highly correlated biomarkers, with a correlation coefficient of ρ > 0.5 (Supplementary Figure S2a,b). TMB had a larger effect size than the neoantigen burden. Hence, neoantigen burden was removed as a biomarker from the MOTIscore. Thus, our final list included the following four biomarkers and their corresponding normalized weights:

- TMB: 0.345
- ICI-Response pathways: 0.208
- TCR repertoire: 0.320
- *PDL1* expression: 0.127

### 3.3. ICI Outcome Prediction and Survival Analysis

**Figure 1:**
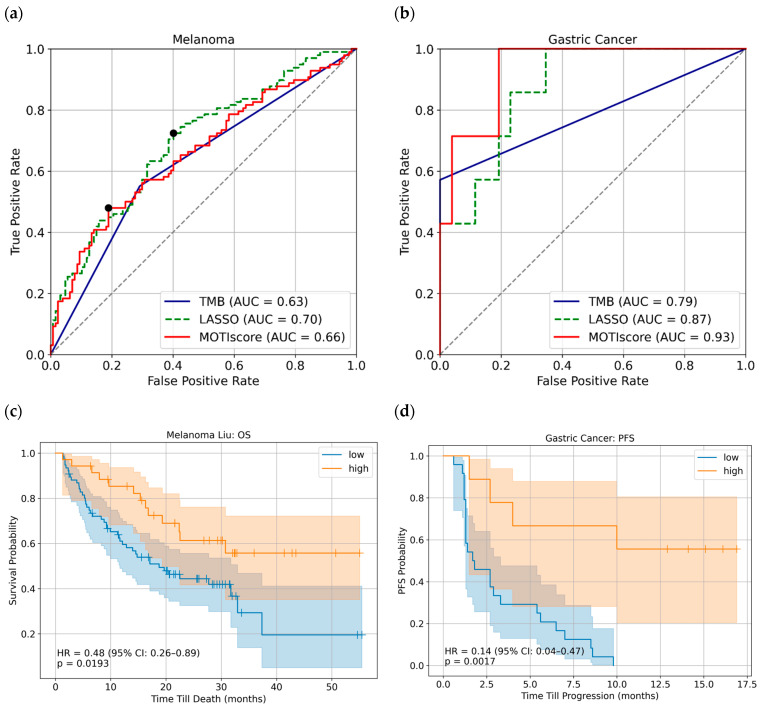
Performance of the MOTIscore and LASSO as predictors of ICI outcomes in (**a**) melanoma and (**b**) gastric cancer. The MOTIscore achieved a higher ROC AUC performance in both groups than TMB alone. The black dot in (**a**) denotes the optimal cutoff, determined using Youden’s index. (**c**) Kaplan–Meier curves of OS in the Liu et al. melanoma cohort. Patients with high MOTIscores experienced better OS, with an HR of 0.48 (95% CI: 0.26–0.89, *p* = 0.0193). (**d**) Kaplan–Meier curves of PFS in the Kim et al. gastric cancer samples. Patients with high MOTIscores clearly exhibited an extended PFS, with an HR of 0.14 (95% CI: 0.04–0.47, *p* = 0.0017).

We evaluated MOTIscore as a predictor of ICI outcome in the melanoma (*n* = 225) and gastric cancer (*n* = 33) cohorts. First, we determined the performance of MOTIscore using receiver operating characteristic (ROC) curves. In Figure 1a,b, we show the ROCs for both cohorts, with the outcome defined as responders and non-responders. For comparison, we also show the ROC curves of the current gold standard TMB with the FDA-approved cutoff of 10 mutations per megabase [47]. For both cancer types, MOTIscore achieved higher ROC areas under the curve (AUCs) than TMB alone. For melanoma samples, our score achieved an ROC AUC of 0.66 versus 0.63 (TMB), whereas it reached an ROC AUC of 0.93 versus 0.79 (TMB) for gastric cancer patients.

Next, we benchmarked the performance of MOTIscore against a machine learning LASSO model, the methodology of which has recently been described [9]. The LASSO model was trained on the melanoma cohort (*n* = 225) using the same set of biomarkers as used for MOTIscore. The scores of both models were highly correlated in both the ICI melanoma and ICI gastric cancer cohorts (Supplementary Figure S3). The ROC curves of the LASSO model are shown in green in Figure 1a,b for the melanoma (*n* = 225) and gastric cancer (*n* = 33) cohorts, respectively. With ROC AUCs of 0.70 for melanoma and 0.87 for gastric cancer, both the LASSO model and the MOTIscore achieved similar results. However, the MOTIscore achieved a higher ROC AUC in the gastric cancer cohort, indicating good generalizability from melanoma to gastric cancer. It is important to note that the LASSO and MOTIscore ROC AUCs for melanoma were calculated using their training cohorts, likely leading to an overestimation of the ROC AUCs in melanoma due to overfitting.

Next, we determined a cutoff for the MOTIscore in the melanoma cohort by maximizing Youden’s index, ensuring an ideal tradeoff between sensitivity and specificity [48]. The cutoff was found to be 0.540, as indicated by the black dot in Figure 1a. Subsequently, we used this threshold to stratify our data for the analysis of PFS and OS. OS data were available for the Liu et al. sub-cohort of melanoma (*n* = 111), and PFS data were available for the Kim et al. gastric cancer cohort (*n* = 33). MOTIscore clearly discriminated responders from non-responders, with a 12-month OS rate of 85% versus 61% in the Liu et al. melanoma cohort, and a 12-month PFS rate of 56% versus 0% in the gastric cancer cohort. The corresponding Kaplan–Meier estimators are shown in Figure 1c,d. A Cox model analysis revealed hazard ratios (HR) of 0.48 (*p* = 0.0193) for melanoma, and of 0.14 (*p* = 0.0017) for gastric cancer, indicating a much lower relapse risk for patients with high MOTIscores.

**Figure 2:**
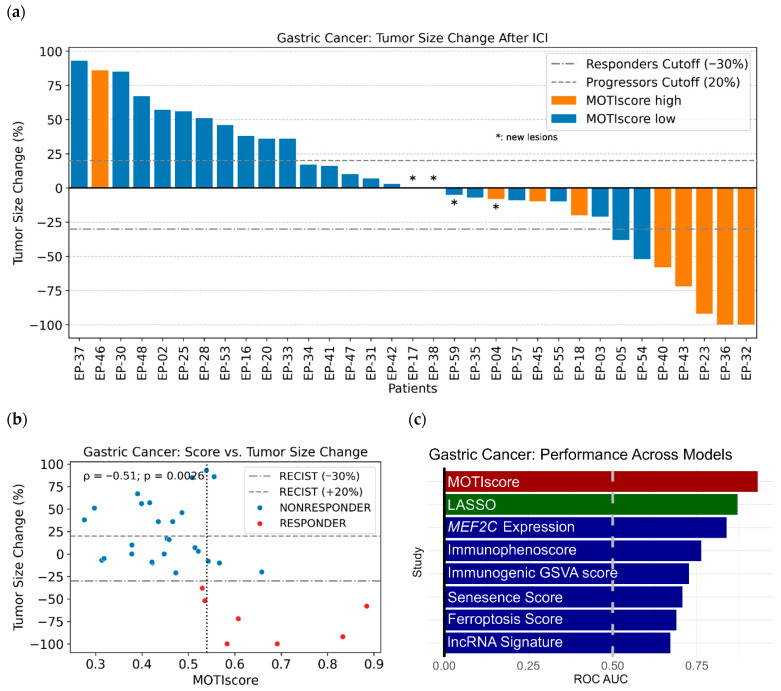
(**a**) Maximal tumor size changes in gastric cancer patients visualized as a waterfall plot. The dotted lines indicate the RECIST classification cutoff values. Bars in orange indicate patients with high MOTIscores whereas blue bars denote patients with low scores. (**b**) Scatter plot of the MOTIscore against the changes in tumor size. The score was inversely correlated with the change in tumor size (Pearson ρ = −0.51 and *p* = 0.0026). The data used to generate this figure were extracted from [46]. (**c**) ROC AUCs of different models predicting ICI outcomes in the Kim et al. gastric cancer cohort. The MOTIscore (red) and the LASSO model (green) exhibited a higher performance than the RNA-based single-omics studies we found (blue).

A subsequent analysis of the maximum tumor size reduction in gastric cancer patients (*n* = 33) after treatment showed additional predictive value of the MOTIscore, Figure 2a. MOTIscores and the corresponding molecular and clinical features are provided in Supplementary Table S2 for the Kim et al. gastric cancer cohort. Of the nine gastric cancer patients with high MOTIscores, eight patients experienced a reduction in tumor size, although not all were classified as responders according to the RECIST cutoffs. MOTIscore was significantly inversely correlated with the change in tumor size after treatment, with a Spearman correlation coefficient of ρ = −0.51 and *p* = 0.0026 (Figure 2b). This underscores the predictive power of our score beyond the “official” RECIST cutoffs.

### 3.4. Benchmarking MOTIscore Against Existing Scores

The gastric cancer cohort (*n* = 33) was used for method benchmarking in various publications. Several authors have evaluated their models using ROC AUC curves, making their results comparable to those of our MOTIscore benchmark. We conducted a paper search using Google Scholar with the search term ‘“PRJEB25780” and “ROC”’ (http://scholar.google.com, accessed on 12 March 2025) to find existing works. After reviewing the search results, we identified six studies that used the Kim et al. gastric cancer cohort and evaluated their models using ROC AUCs, with the outcome defined in terms of RECIST [49,50,51,52,53,54]. In contrast to our multi-omics approach, all other models were based on single-omics RNA-Seq data. The methodologies used in the existing studies varied widely. Two models were designed to leverage expression of immunogenicity genes, one model was based on the expression of senescence genes, and one model on the expression of ferroptosis genes. One study made predictions based on *MEF2C* expression. Finally, one study made predictions using a long non-coding RNA (lncRNA) signature.

Figure 2c shows a bar plot comparing the ROC AUCs of these studies with MOTIscore. ROC AUCs of RNA-based models ranged from 0.67 to 0.84, with a median value of 0.72. MOTIscore outperformed the RNA-based models with an ROC AUC of 0.93. Note that the effective cohort size of the benchmark tests varies, as the quality control filters of the studies may vary, and because we could only utilize cases having both DNA and RNA-Seq.

### 3.5. Gene Set Enrichment Analysis

**Figure 3:**
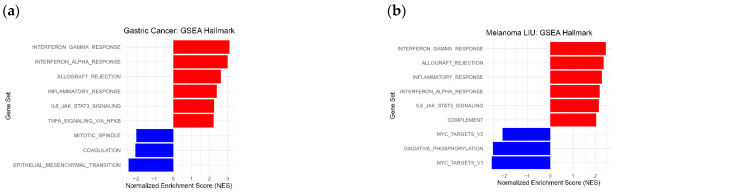

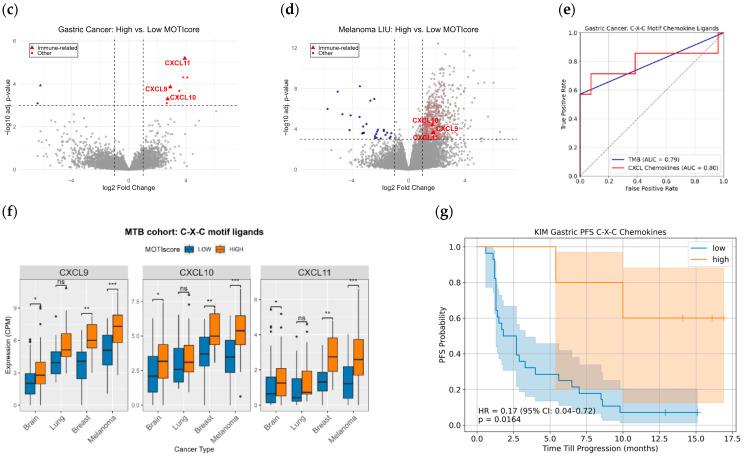
(**a**,**b**) GSEA results for the gastric cancer (*n* = 33) and the Liu et al. melanoma (*n* = 111) cohorts using the Hallmark gene sets. Immune-related pathways were enriched in the MOTIscore-high group, with significance thresholds of NES ≥ 2 and of adjusted *p* < 0.001. Enriched pathways are indicated in red, whereas depleted pathways are blue. (**c**,**d**) Volcano plots of the gastric cancer cohort and the Liu et al. melanoma cohort, with the MOTIscore as the group discriminator. Many immune-related genes (indicated by triangles (▲)) were significantly up-regulated. We used significance cutoffs of *p* < 0.001 and a log_2_ fc ≥ 1. Up-regulated genes are denoted in red, down-regulated genes in blue, and non-significant genes in gray. (**e**) The combined expression of *CXCL9*, *CXCL10*, and *CXCL11* was predictive of ICI outcomes in gastric cancer (*n* = 33), with an ROC AUC of 0.80. (**f**) Distribution of the C-X-C motif chemokine ligands expression across cancer types in the MTB cohort. Significance levels are denoted with asterisks (ns: non-significant, *: *p* < 0.05, **: *p* < 0.01, ***: *p* < 0.001), and outliers are represented with black dots. (**g**) Kaplan–Meier curves for this combined biomarker. Patients with high levels exhibited a significantly extended PFS in gastric cancer, with an HR of 0.17 (*p* = 0.0164).

Next, we conducted an analysis using DESeq2 to identify differentially expressed genes (DEGs) and pathways enriched for DEGs in the RNA-Seq samples. We stratified patients into two groups with high and low MOTIscores. Based on the detected DEGs, we performed GSEA for the Kim et al. gastric cancer cohort (*n* = 33) using the MSigDB Hallmark gene sets [55]. This analysis revealed several enriched immune-related gene sets in the group with high MOTIscores (Figure 3a). After applying significance thresholds (adjusted *p* < 0.001 and normalized enrichment score (NES) ≥ 2), the following six Hallmark gene sets were enriched in gastric cancer: interferon gamma response, interferon alpha response, allograft rejection, inflammatory response, *IL6* JAK *STAT3* signaling, and *TNFA* signaling via NF-κB. All enriched gene sets describe immune system related processes.

For melanoma, we performed another GSEA using the Liu et al. melanoma ICI sub-cohort (*n* = 111). Six significantly enriched Hallmark gene sets were identified in the group with high MOTIscores: interferon gamma response, allograft rejection, inflammatory response, interferon alpha response, *IL6* JAK *STAT3* signaling, and complement system (Figure 3b). Similar to gastric cancer, all enriched pathways describe processes of the immune system. The gene sets found in melanoma corresponded to those identified in patients with gastric cancer, with an overlap of four pathways.

### 3.6. C-X-C Motif Chemokine Ligands Predict ICI Outcome and Survival

Many immune-related genes were significantly up-regulated in the group with high MOTIscores, as indicated by the small triangles in the volcano plots for gastric cancer (*n* = 33) and the Liu et al. melanoma cohort (*n* = 111), Figure 3c,d. We defined a gene as immune-related if it occurred in one of the MSigDB Hallmark gene sets describing immune system processes. Importantly, the C-X-C motif chemokine ligands *CXCL9*, *CXCL10*, and *CXCL11* were significantly up-regulated in patients with high MOTIscores in both cancer types, suggesting the presence and activation of immune cells in these tumors. To confirm these findings, we conducted an additional analysis of DEGs in the MTB cohort (*n* = 559), using the MOTIscore as the group discriminator. At least two-fold upregulation of *CXCL9*, *CXCL10*, and *CXCL11* was observed in all cancer types including melanoma (*n* = 77), breast cancer (*n* = 40), brain cancer (*n* = 198), and lung cancer when comparing samples with high to low MOTIscores (Figure S4), with significance for melanoma and breast cancer. Figure 3f shows box plots of the expression distribution of *CXCL9*, *CXCL10*, and *CXCL11* in MTB brain, lung, breast, and melanoma samples, with the samples stratified into MOTIscore-high and MOTIscore-low groups. The median expression of all three genes was lowest in brain and lung cancers, whereas higher expression values were detected in melanoma and breast cancer. We used the Mann–Whitney U test with Benjamini–Hochberg correction to detect significant differences in the distribution between samples with low and high MOTIscores. Significant differences were observed in melanoma, breast, and brain malignancies, whereas no significant differences were observed in lung cancer.

The genes *CXCL9*, *CXCL10*, and *CXCL11* are a crucial components of the C-X-C motif chemokine family, which plays an important role in the attraction and activation of immune cells [56]. Recent studies have suggested predictive relevance of these genes for ICI treatment [57]. Thus, we conducted an exploratory analysis to investigate their effects on treatment outcome in the cohorts used in this study. To investigate the predictive value of *CXCL9*, *CXCL10*, and *CXCL11* for ICI outcomes, we first evaluated each gene individually. We found that the expression of each gene was a modest predictor of ICI outcomes in the ICI melanoma (*n* = 225) and gastric cancer cohorts (*n* = 33), with ROC AUCs ranging from 0.53 to 0.79 (Supplementary Figure S5a,b). We then merged the genes into a single biomarker using the mean value of their log_2_ fc gene expression. This combined C-X-C motif chemokine expression marker was predictive of ICI outcomes in terms of both ROC AUCs and survival. In gastric cancer (*n* = 33), the combined biomarker achieved an ROC AUC of 0.80, thereby outperforming TMB (Figure 3e). In the ICI melanoma cohort (*n* = 225), we observed an ROC AUC of 0.57, which was better than random but was outperformed by TMB (Supplementary Figure S5c).

We determined a cutoff value of this combined biomarker by maximizing Youden’s index in the melanoma ICI cohort (*n* = 225). The ICI cohorts were then stratified into two groups based on high and low C-X-C- motif chemokine ligand expression. Figure 3g shows the corresponding Kaplan–Meier curve for the gastric cancer cohort (*n* = 33). Cox model analysis revealed a significantly (HR = 0.17, *p* = 0.0164) extended PFS in patients with highly expressed C-X-C motif chemokine ligands. The Kaplan–Meier curves for the Liu et al. melanoma cohort (*n* = 111) suggest an extended OS in melanoma (HR = 0.72), but this was non-significant (*p* = 0.2240), Supplementary Figure S5d.

### 3.7. MOTIscore Distribution Across Cancer Types and Clinical Significance in MTB Cohort

In this section, we investigate the distribution of the MOTIscore within the heterogeneous pan-cancer MTB cohort (*n* = 559). All patients were sequenced between 2020 and 2024 at the local MTB and received various anti-cancer drugs [23]. This MTB cohort was highly heterogeneous; most tumors were advanced, and patients had received multiple diverse cancer therapies. The most abundant cancer types were malignancies affecting the brain (*n* = 198, ICD10 C70 and C71), melanoma (*n* = 77, ICD10 C43), breast (*n* = 40, ICD10 C50), and lung (*n* = 29, ICD10 C34). All other cancer types present in the MTB cohort had <25 samples and were summarized as “other” (*n* = 215). A complete description of the cohort is provided in Supplementary Table S1.

**Figure 4:**
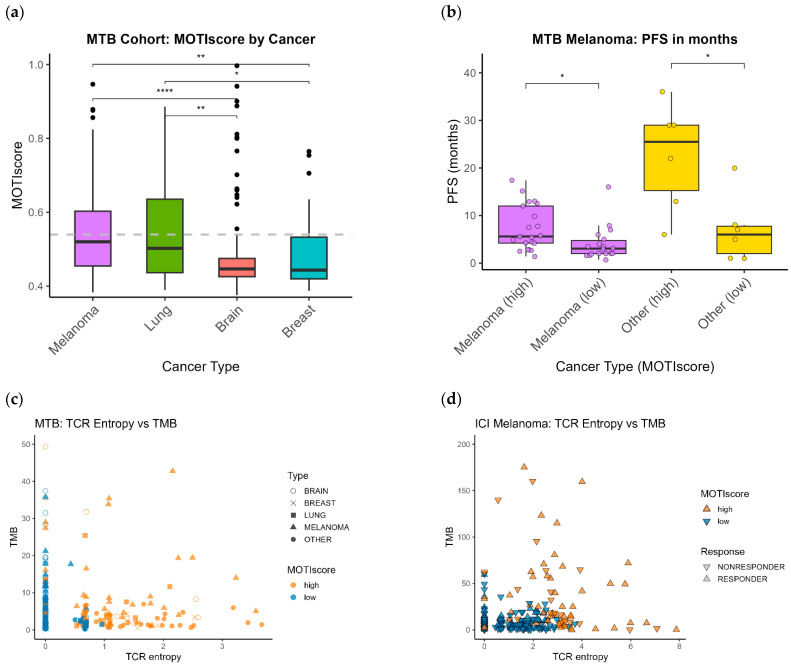
(**a**) Distribution of MOTIscores in cancer entities with more than 25 samples in the MTB cohort. Kruskal–Wallis test followed by Dunn’s test with Benjamini–Hochberg correction revealed significant differences in the score distributions. The dashed line depicts the cutoff value of 0.540, which was determined using Youden’s index. (**b**) ICI-specific PFS of MTB melanoma (*n* = 39) and other cancer types (*n* = 12) for which ICI duration was available. Patients with high MOTIscores exhibited significantly higher ICI-specific PFS than those with low MOTIscores did. The asterisks denote significance levels (*: *p* < 0.05, **: *p* < 0.01, ****: *p* < 0.0001) (**c**) Scatter plot of TCR repertoire versus TMB in the pan-cancer MTB cohort (*n* = 559). Orange symbols indicate samples with high MOTIscores, whereas blue symbols represent samples with low MOTIscores. The cancer type is denoted by shape. (**d**) TCR repertoire versus TMB in the ICI melanoma cohort (*n* = 225). Responders are denoted with ▲, and non-responders with ▼.

Figure 4a shows boxplots of the MOTIscore for the most abundant cancer types in the MTB cohort. The distribution of the scores varied widely across cancer types. The highest median MOTIscores were found in melanoma and lung cancer, whereas brain and breast cancers showed a very low median score. A Kruskal–Wallis test revealed significant differences in the MOTIscore distributions between melanoma and lung (higher scores) versus brain and breast (lower scores), Figure 4a. Moreover, brain cancer showed the lowest overall distribution of scores (lowest 75% quantile). The median MOTIscores of all cancer types were below Youden’s cutoff of 0.540, highlighting the potential use of the MOTIscore in identifying few patients who may benefit from ICI. In both melanoma and lung cancer, approximately 45% of patients had a high MOTIscore, matching previously published estimates of patients potentially responsive to ICI in these cancer types [58]. In breast cancer, less than a quarter of patients showed a high MOTIscore. However, these patients might be of particular interest for the selection of ICIs, even if immunotherapy is not part of the standard treatment for this cancer entity (Figure 4a and Supplementary Table S1). For brain cancer, we only observed a few outliers above the threshold, which was expected, as brain has low immunogenicity. We speculate that in the outlier patients, with sometimes very high MOTIscore, the blood–brain barrier is compromised, allowing immune cells to enter in large amounts. Furthermore, glioblastomas pre-treated with a chemotherapy often exhibit hypermutation, leading to a very high TMB [59].

A total of 52 patients in the MTB cohort received ICI treatment. ICI treatments were usually stopped due to disease progression, but it remains unclear whether these endpoints were censored or whether progression occurred. Thus, we termed this duration the ICI-specific PFS. ICI-specific PFS in melanoma (*n* = 39) and other cancer types (*n* = 12) was extended in patients with high MOTIscores, Figure 4b. The effect was large, with effect sizes of 0.8 in melanoma and of 1.7 in the other cancer entities, as was determined using Cohen’s d. The extended ICI-specific PFS was significant (*p* < 0.05), as proven using the Mann–Whitney U test with Benjamini–Hochberg correction for multiple hypothesis testing.

### 3.8. Clinical Significance of the TCR Repertoire Biomarker

The two main contributing biomarkers to the MOTIscore (highest weights) were TCR repertoire and TMB. Samples with high TCR repertoire were likely classified as MOTIscore-high. In contrast, high TMB values with low TCR entropy did not necessarily lead to high MOTIscores in the MTB pan-cancer and melanoma cohorts. In Figure 4c,d, we show scatter plots of TMB versus TCR repertoire in the MTB pan-cancer (*n* = 559) and the ICI melanoma cohorts (*n* = 225). MOTIscore-high samples are shown in orange, whereas MOTIscore-low samples are denoted in blue. Figure 4c revealed mainly melanoma, but also brain, lung, and other types of tumor samples showing concomitant MOTIscore-high and high TCR repertoire in the MTB cohort (*n* = 559). These patients may benefit from and should be considered for ICI therapy as part of a personalized treatment strategy. Indeed, most responders in the ICI melanoma cohort (*n* = 225), Figure 4d, had concomitant high TCR entropy and MOTIscore.

Using the ICI melanoma cohort (*n* = 225), we analyzed the proportion of responders depending on the TCR repertoire and TMB. The samples were stratified into four groups based on high and low TCR repertoires, and high and low TMB statuses (Table 1). The fewest responders (25.7%) were found in the group with low TCR repertoire and low TMB. Conversely, the highest proportion of responders (67.9%) was found in the group with both high TMB and TCR repertoire. Interestingly, the group with high TCR repertoire but low TMB also showed an increase in responders (41.7% compared to 25.7%), supporting our suggestion to use TCR repertoire as part of the MOTIscore and as an independent biomarker for selecting patients for ICI, even at low TMB status. Hence, our findings provide further evidence that integrating RNA- and DNA biomarkers can help identify patients who may benefit from ICI treatment.

## 4. Discussion

The multi-omics tumor immunogenicity score outperformed TMB in both melanoma and gastric cancers, as demonstrated by better ROC AUCs, Figure 1a,b. Furthermore, we showed that RNA-derived biomarkers, such as TCR repertoire and C-X-C- motif chemokine ligand expression, discriminate between responders and non-responders and are predictive of PFS and OS in some cancer types. These findings confirm the hypothesis that integrating multiple predictive biomarkers derived from both DNA and RNA sequencing can lead to improved overall predictions compared with the use of single biomarkers as predictors. This was also evident in our analysis of the ICI melanoma cohort, in which the proportion of responders was highest in patients with high TMB and TCR repertoire (Table 1). However, MOTIscore only moderately improved predictions in terms of ROC AUCs. Nonetheless, additional analyses (Figure 1c,d and Figure 4b) showed that patients with high MOTIscore had significantly longer PFS and a higher chance of strong shrinkage of the tumor size. Moreover, our analysis demonstrated that MOTIscore generalizes well to different cancer types. In patients with melanoma, those with high MOTIscores exhibited improved OS and ICI-specific PFS. Similarly, in patients with gastric cancer, high MOTIscores were associated with an extended PFS and high probability of tumor shrinkage. These results underscore the potential use of MOTIscore as a decision support tool for ICI therapy in clinical settings.

The MOTIscore is straightforward to calculate and provides transparent results by design. As WES and RNA-Seq data become increasingly available in routine clinical oncology, our score can be calculated as part of standard genetic tumor diagnostics. No additional diagnostic tests are necessary because the MOTIscore only leverages molecular biomarkers derived from NGS data. However, potential clinical applications will require validation studies in prospective studies with separate cancer entities. The MOTIscore could help to select patients with high chances of success for ICI treatment. Our approach can easily be extended by simply adding and reweighting additional molecular features if additional biomarkers are of interest or are discovered. For instance, the expression of C-X-C chemokine ligands discovered in this study could be added as a feature to the MOTIscore in a prospective study.

The GSEAs of melanoma and gastric cancer revealed intersecting immunogenicity gene sets that were enriched in both cancers, Figure 3a,b. This overlap might indicate that the MOTIscore captures the general underlying molecular tumor properties, leading to the aforementioned good generalizability of the score. The C-X-C motif chemokine ligand genes *CXCL9*, *CXCL10*, and *CXCL11* were up-regulated in patients with high MOTIscores in gastric cancer and melanoma (Figure 3c,d), and we confirmed this finding for different cancer types in the independent MTB cohort (Figure 3f). Consistent with the role of the C-X-C motif pathway in immune cell activation and attraction [56], we showed that the expression of these three genes was a good predictor of ICI outcomes and survival in both melanoma and gastric cancer (Figure 3e,g and Supplementary Figure S4).

For gastric cancer, we compared the predictions of MOTIscore with those of existing models (Figure 2c). All benchmark studies we identified by the literature search applied single-omics models based only on RNA-Seq, whereas MOTIscore and our LASSO model were based on both WES and RNA-Seq. Both multi-omics models outperformed single-omics models in predicting ICI outcomes. This finding suggests that integrating various multi-omics biomarkers provides a more holistic characterization of tumor immunogenicity, thereby leading to better ICI outcome predictions [60,61]. However, we could only compare the outcomes with respect to RECIST, but not survival (PFS or OS), as survival analysis was not available for the other studies.

Our pan-cancer analysis using the local MTB cohort revealed distinct MOTIscore distributions across cancer types, particularly between immunogenic tumors and tumors that are normally considered to have low immunogenicity (Figure 4a). This showed that the MOTIscore also reflects molecular immunogenic properties at the level of cancer types, reflecting cancer type-specific treatment strategies. The variation in the MOTIscore distributions aligns well with the immunologic classification of tumors as “hot” (e.g., melanoma, lung and gastric cancer) and “cold” (e.g., glioblastoma, meningioma, and breast cancer) [62]. MOTIscore may help identify hot tumors, even in typically “cold” cancer types, because it integrates several properties frequently used to distinguish hot and cold tumors, such as TMB and tumor-infiltrating lymphocytes. For instance, 13 of 40 of the MTB breast cancer patients had high MOTIscores (>0.54), suggesting a potential clinical benefit for these patients from ICI, although breast cancer is normally considered a cold tumor type [63].

We conducted a separate analysis of the TCR repertoire and TMB, Figure 4c,d and Table 1, because these two biomarkers contributed the most to the MOTIscore with weights > 0.3. Our in-depth analysis showed that both biomarkers were predictive of ICI outcomes in melanoma, Table 1. The poorest outcomes were observed in patients with both a low TCR repertoire and TMB, but interestingly, the group of patients with low TMB but high TCR repertoire had a strongly increased fraction of responders. Hence, TCR repertoire should be used in addition to TMB as a criterion for selecting patients for ICI therapy. Other recent studies have similarly demonstrated the independent predictive performance of genomic and transcriptomic biomarkers in melanoma [9].

The neoantigen burden was highly correlated with TMB and was subsequently excluded from the MOTIscore (Supplementary Figure S2). This finding confirms that TMB can be used as a proxy for neoantigen burden [64]. However, recent studies have suggested that the immune response is mostly driven by very few or a single high-quality neoantigens [65]. Thus, the contribution of individual neoantigens could be a better predictor of anti-tumor immunogenicity than neoantigen burden or TMB. By focusing only on TMB, we might underestimate the immunogenicity of tumors with low TMB, in which only a few highly potent neoantigens drive anti-tumor immunogenicity. Conversely, we could easily overestimate immunogenicity in tumors with high TMB in which no immunogenic antigens were present. Future studies should include features describing neoantigen quality rather than quantity, with an appropriate neoantigen prioritization algorithm [66].

Our study had several other limitations. First, some biomarkers were sparsely enriched in the melanoma discovery cohort. For instance, we detected only two cases with alterations in the ICI-resistance pathways. These two melanoma cases exhibited homozygous deletions in *B2M*, a strong predictor of ICI-resistance, and both were indeed non-responders with progressive disease. However, ICI-resistance mutations were rejected as biomarkers because they were not significantly associated with ICI outcomes due to their rarity. Hence, a larger discovery cohort would likely lead to a higher number of significant biomarkers that could be included in MOTIscore. An important limitation was our restriction to samples with a tumor purity >30%, although purity is also a molecular tumor property relevant for ICI efficacy [67]. Therefore, this filter may have led to bias. Apart from molecular genetic data, we did not incorporate additional clinical data into MOTIscore, limiting its applicability to certain patients. For example, patients with brain metastases may not benefit from ICI despite high MOTIscores, as we observed in one MTB melanoma patient with brain metastasis. Given the nature of the available data, it remains unclear whether MOTIscore was predictive of ICI treatment or whether it was just a general prognostic marker. However, this problem can only be addressed using a specific prospective study design that involves a non-ICI control group before data acquisition.

## 5. Conclusions

In summary, we showed that multi-omics biomarkers derived from exome and transcriptome sequencing can be integrated into a unified score, leading to improved characterization of tumor immunogenicity. Comparative analyses of samples with high versus low MOTIscores revealed enrichment of pathways implicated in the immune regulation of tumors, such as the C-X-C chemokine signaling pathway. The predictive performance of the MOTIscore was comparable to that of several other models. MOTIscore demonstrated robust accuracy in forecasting immune checkpoint inhibitor (ICI) responses according to the RECIST criteria, outperforming single-biomarker approaches. High MOTIscores, particularly in cases in which both TCR diversity and TMB were high, were associated with prolonged overall and progression-free survival in patients with melanoma and gastric cancer who were treated with ICIs, as well as with an increased chance of a strongly reduced tumor size in gastric cancer. These findings suggest that the MOTIscore may serve as a clinically useful tool for identifying patients who are likely to benefit from ICI therapy. Hence, it potentially supports treatment prioritization in molecular tumor boards, especially after validating the findings presented here in a prospective study. Thereby, MOTIscore advances personalized oncology.

## Figures and Tables

**Table 1:** We stratified the patients in the ICI melanoma cohort (*n* = 225) into four groups depending on their TMB and TCR repertoire levels. Both biomarkers were predictive of the outcomes. However, most responders were found in the group with both a high TMB and TCR repertoire. Conversely, the response rate was the lowest in patients with low levels of both biomarkers.

TCR Repertoire	TMB	Non-Responders	Responders	Response Rate
Low	Low	55	19	25.7%
High	Low	35	25	41.7%
Low	High	18	20	52.6%
High	High	17	36	67.9%

## Data Availability

Most of the data used in this study were obtained from publicly available NGS datasets. These data can be obtained from the European Nucleotide Archive (ENA), the database of Genotypes and Phenotypes (dbGaP), or the Gene Expression Omnibus (GEO). The melanoma cohort was assembled from six different studies, with the following study accession numbers: Amato et al.: PRJNA639866, GSE15996 [44]; Hugo et al.: SRP090294, SRP067938 [43]; Liu et al.: phs000452.v3.p1 [42]; Pyke et al.: phs002388.v1.p1 [41]; Riaz et al.: SRP094781, GSE91061 [40]; Wolchok et al.: SRP417444 [45]. The gastric cancer dataset was described by Kim et al. [46] and is available under the study accession number PRJEB25780. The PFS data and the tumor reduction data for the gastric cancer cohort were extracted from Figure 1 of that paper. The data of the local MTB patients cannot be shared in a non-anonymized form owing to broad consent restrictions. A minimal working example for the MOTIscore algorithm can be found on our GitHub repository, https://github.com/axelgschwind/MOTIscore/ (accessed 25 November 2025, commit fbe6101).

## Supplementary Materials

The following supporting information can be downloaded at: https://www.mdpi.com/article/10.3390/biology14121698/s1, Figure S1: Outline of the study. First, we determined the weights using a cohort of ICI-treated melanoma patients, who were classified as responders and non-responders. Statistical tests (Mann Whitney U and Fisher’s exact test) provided *p*-values that served as weights for our MOTIscore. Biomarkers that exhibited high mutual correlation coefficients were removed. The MOTIscore was subsequently calculated using z-scores for each biomarker. Finally, the results were evaluated using three distinct cohorts; Figure S2: (a) Heatmap of Spearman’s rank correlation coefficients between all biomarkers that were discriminative for ICI outcomes in the melanoma cohort, with significance levels *p* < 0.05. (b) Only TMB and Neoantigen Burden were highly correlated, with a Spearman’s rank correlation coefficient of > 0.5. Consequently, we removed the Neoantigen Burden as a biomarker for our score. The correlations were calculated using the melanoma discovery cohort, which was used to determine the weights for the MOTIscore; Figure S3: Correlation of MOTIscore and LASSO regression scores in (a) melanoma (*n* = 225) and (b) gastric cancer (*n* = 33). Both scores were highly correlated, with Pearson’s correlation coefficients close to 1 in both cohorts; Figure S4: Volcano plots for the (a) MTB melanoma (*n* = 77), (b) MTB brain cancer (*n* = 198), (c) MTB lung cancer (*n* = 29), and (d) MTB breast cancer (*n* = 40) cohorts. All three C-X-C motif chemokine ligands *CXCL9*, *CXCL10*, and *CXCL11* were significantly enriched in melanoma, and *CXCL9* was up-regulated in brain cancer. However, none of these genes was enriched in lung or breast cancer; Figure S5: ROC AUCs of *CXCL9*, *CXCL10*, and *CXCL11* expression in (a) the ICI melanoma cohort (*n* = 225) and (b) the gastric cancer cohort (*n* = 33). All three genes were predictive of ICI outcomes in both cancer types. In gastric cancer, they achieved performance similar to that of TMB. (c) The combination of the log2 fc of these three genes was better than random in the ICI melanoma cohort (*n* = 225) but was outperformed by TMB. (d) The Kaplan-Meier curves did not show significant differences in terms of OS in the Liu et al. melanoma cohort (*n* = 111); Table S1: MTB Cohort Overview; Table S2: MOTIscores Gastric Cancer.
