# Supplementary material for: Multi-Omics Tumor Immunogenicity Score Predicts Immunotherapy Outcome and Survival
Source: Biology (Basel). 2025 Nov 28;14(12):1698. doi: 10.3390/biology14121698 (PMC12730895; doi:10.3390/biology14121698)
Supplement: Supplementary file 1 [file biology-14-01698-s001.zip › Supplementary_Figures.pdf]

Supplementary Figures

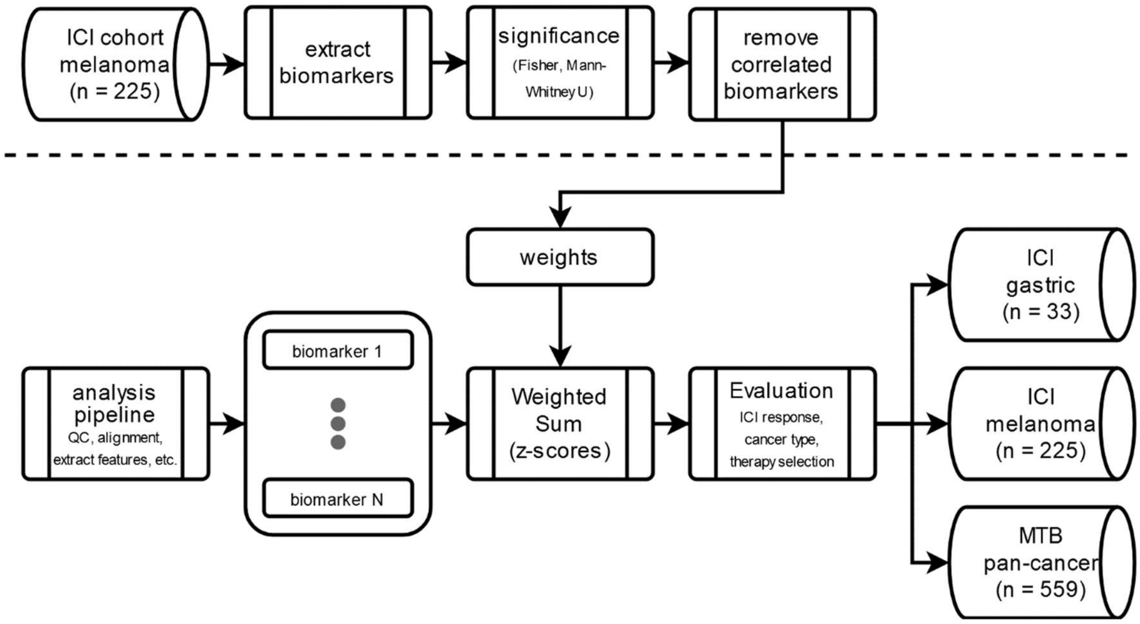

Figure S1. Outline of the study. First, we determined the weights using a cohort of ICI-treated melanoma patients, who were classified as responders and non-responders. Statistical tests (Mann Whitney U and Fisher's exact test) provided p-values that served as weights for our MOTIscore. Biomarkers that exhibited high mutual correlation coefficients were removed. The MOTIscore was subsequently calculated using z-scores for each biomarker. Finally, the results were evaluated using three distinct cohorts.

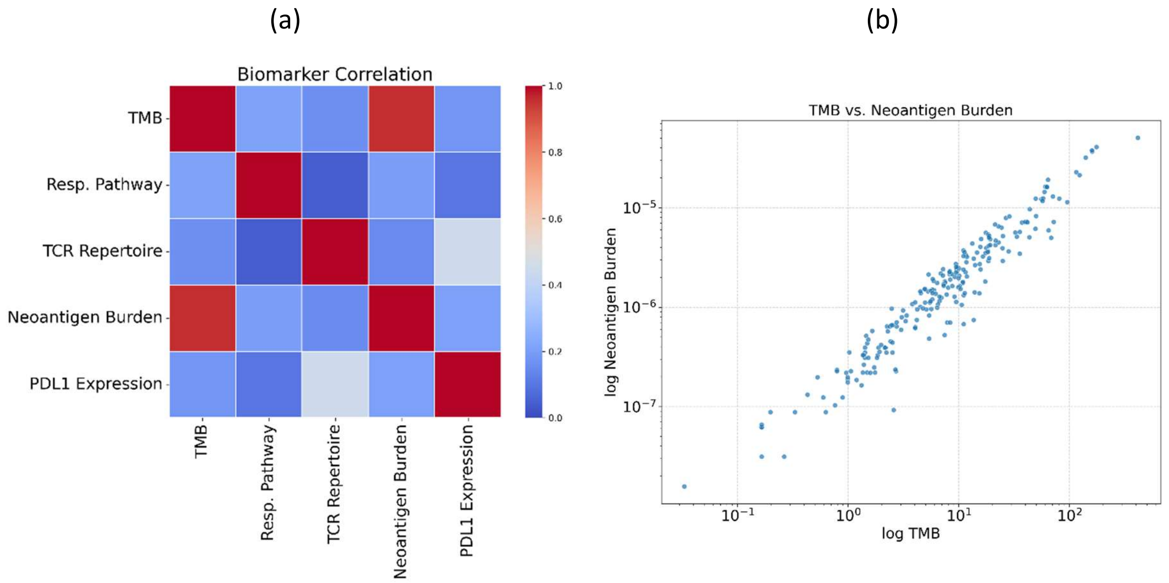

Figure S2. (a) Heatmap of Spearman's rank correlation coefficients between all biomarkers that were discriminative for ICI outcomes in the melanoma cohort, with significance levels $p < 0.05$. (b) Only TMB and Neoantigen Burden were highly correlated, with a Spearman's rank correlation coefficient of > 0.5. Consequently, we removed the Neoantigen Burden as a biomarker for our score. The correlations were calculated using the melanoma discovery cohort, which was used to determine the weights for the MOTIscore.

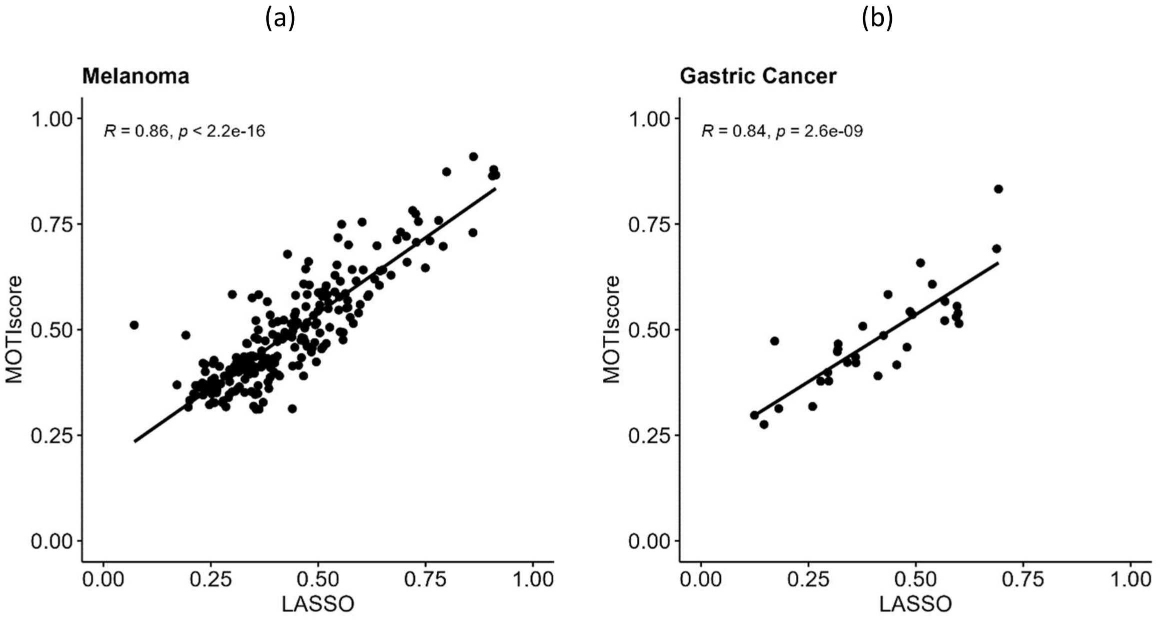

Figure S3. Correlation of MOTIscore and LASSO regression scores in (a) melanoma (n = 225) and (b) gastric cancer (n = 33). Both scores were highly correlated, with Pearson's correlation coefficients close to 1 in both cohorts.

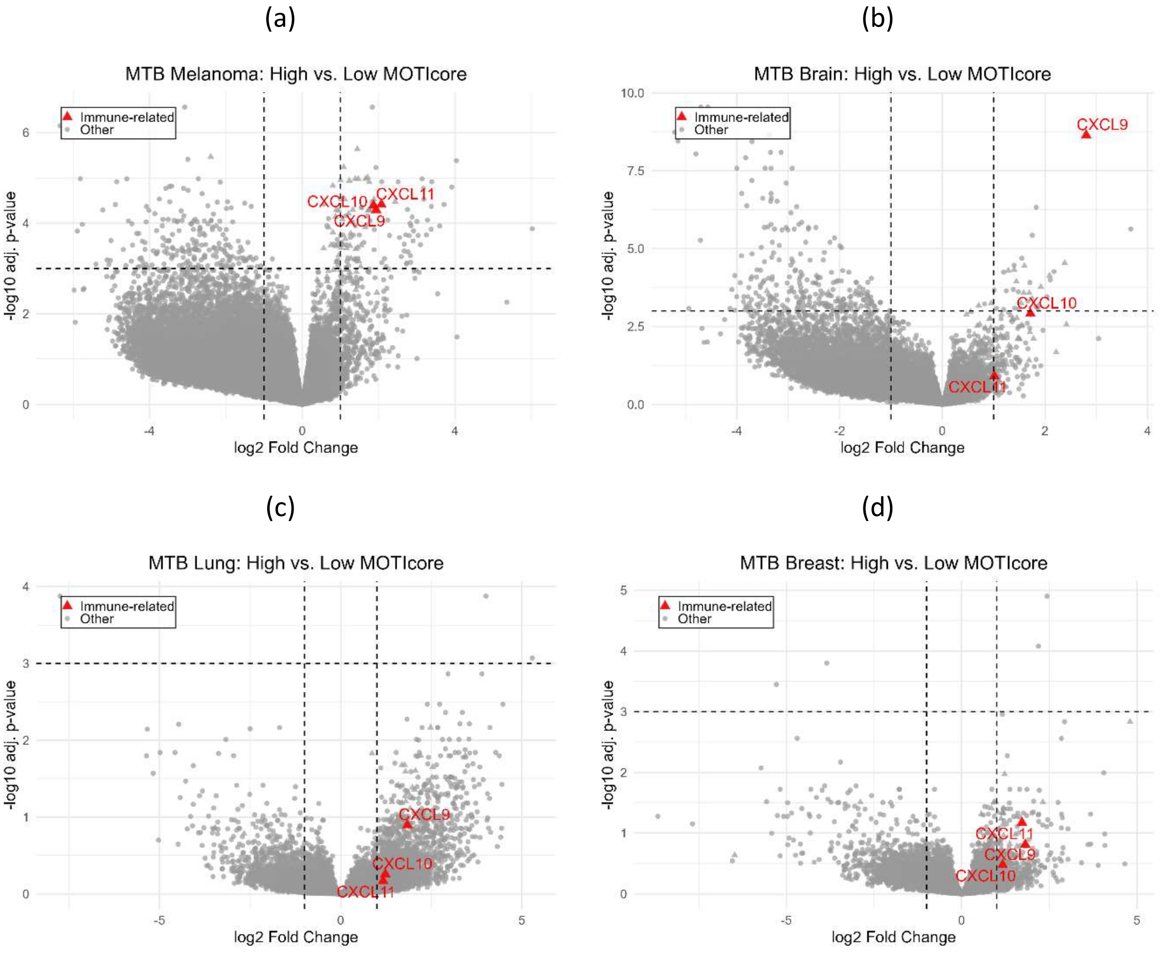

Figure S4. Volcano plots for the (a) MTB melanoma (n = 77), (b) MTB brain cancer (n = 198), (c) MTB lung cancer (n = 29), and (d) MTB breast cancer (n = 40) cohorts. All three C-X-C motif chemokine ligands CXCL9, CXCL10, and CXCL11 were significantly enriched in melanoma, and CXCL9 was up-regulated in brain cancer. However, none of these genes was enriched in lung or breast cancer.

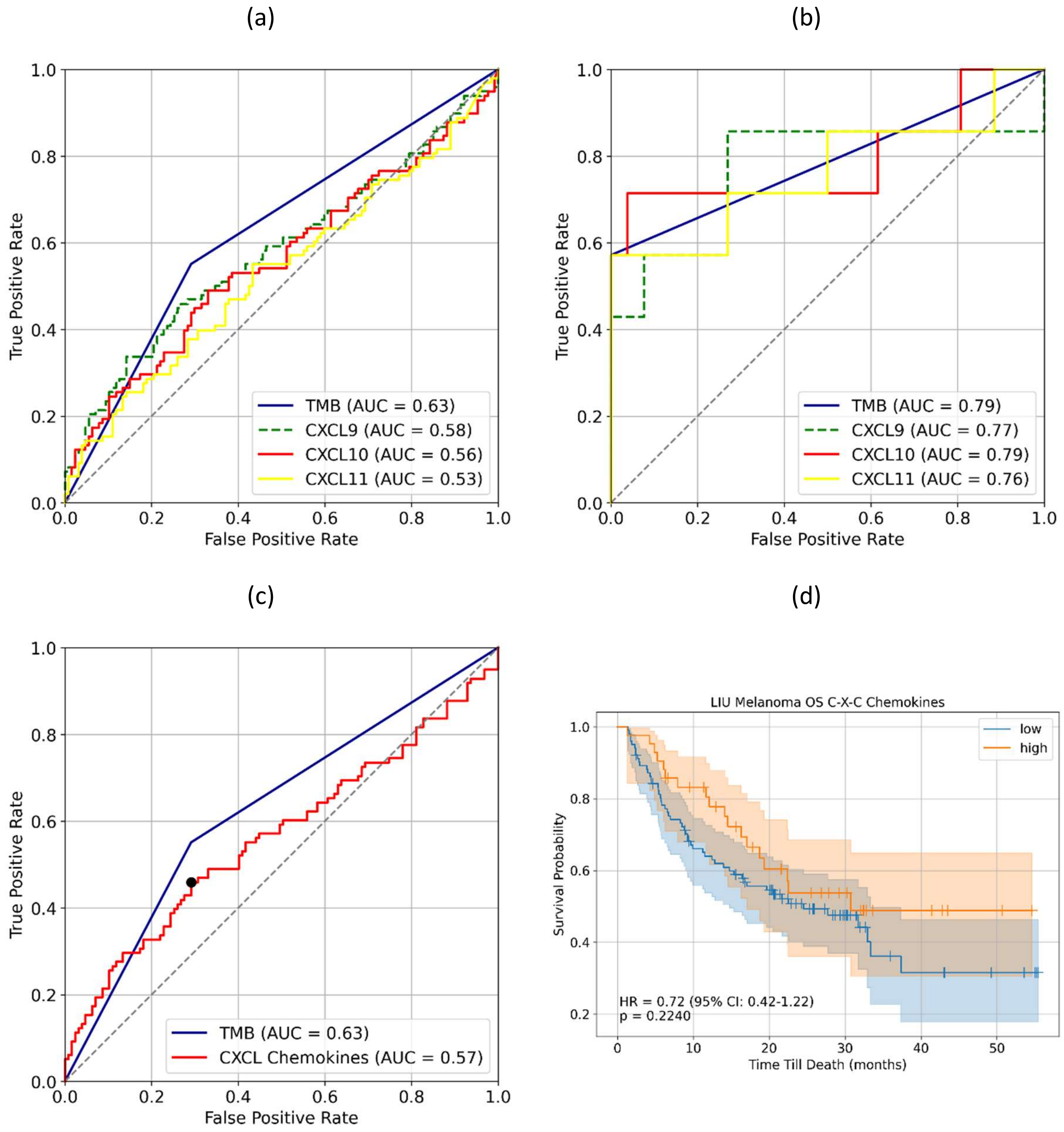

Figure S5. ROC AUCs of CXCL9, CXCL10, and CXCL11 expression in (a) the ICI melanoma cohort (n = 225) and (b) the gastric cancer cohort (n = 33). All three genes were predictive of ICI outcomes in both cancer types. In gastric cancer, they achieved performance similar to that of TMB. (c) The combination of the log2 fc of these three genes was better than random in the ICI melanoma cohort (n = 225) but was outperformed by TMB. (d) The Kaplan-Meier curves did not show significant differences in terms of OS in the Liu et al. melanoma cohort (n = 111).
